# Boosting the Oxygen Evolution Reaction by Controllably Constructing FeNi_3_/C Nanorods

**DOI:** 10.3390/nano12152525

**Published:** 2022-07-22

**Authors:** Xu Yu, Zhiqiang Pan, Zhixin Zhao, Yuke Zhou, Chengang Pei, Yifei Ma, Ho Seok Park, Mei Wang

**Affiliations:** 1 School of Chemistry and Chemical Engineering, Yangzhou University, Yangzhou 225009, China; w461015600@163.com (Z.P.); zhao1zhi2xin3@163.com (Z.Z.); zykds66@163.com (Y.Z.); chengpyzu@163.com (C.P.); 2 State Key Laboratory of Quantum Optics and Quantum Optics Devices, Institute of Laser Spectroscopy, Collaborative Innovation Center of Extreme Optics, Shanxi University, Taiyuan 030006, China; mayifei@sxu.edu.cn; 3 Department of Chemical Engineering, College of Engineering, Sungkyunkwan University, 2066 Seobu-ro, Jangan-gu, Suwon-si 440-746, Gyeonggi-do, Korea; phs0727@skku.edu

**Keywords:** FeNi_3_ alloy, nanorods, bimetallic, oxygen evolution reaction

## Abstract

Transition bimetallic alloy-based catalysts are regarded as attractive alternatives for the oxygen evolution reaction (OER), attributed to their competitive economics, high conductivity and intrinsic properties. Herein, we prepared FeNi_3_/C nanorods with largely improved catalytic OER activity by combining hydrothermal reaction and thermal annealing treatment. The temperature effect on the crystal structure and chemical composition of the FeNi_3_/C nanorods was revealed, and the enhanced catalytic performance of FeNi_3_/C with an annealing temperature of 400 °C was confirmed by several electrochemical tests. The outstanding catalytic performance was assigned to the formation of bimetallic alloys/carbon composites. The FeNi_3_/C nanorods showed an overpotential of 250 mV to afford a current density of 10 mA cm^−2^ and a Tafel slope of 84.9 mV dec^−1^, which were both smaller than the other control samples and commercial IrO_2_ catalysts. The fast kinetics and high catalytic stability were also verified by electrochemical impendence spectroscopy and chronoamperometry for 15 h. This study is favorable for the design and construction of bimetallic alloy-based materials as efficient catalysts for the OER.

## 1. Introduction

There is a global consensus that producing hydrogen energy via electrochemical water splitting will lighten the burden of consuming energy from fossil fuels and replace unsustainable energy sources [1,2,3,4,5]. The oxygen evolution reaction (OER) is a half-reaction of the electrolysis of water, but the issue of slow reaction kinetics during the complicated four-electron transfer process critically needs to be solved [6,7]. The energy conversion efficiency of catalysts for practical water splitting is affected by the high overpotential and energy consumption of catalysts during the OER process [8,9]. Ruthenium dioxide (RuO_2_) and iridium dioxide (IrO_2_), as effective OER catalysts [10,11,12], can facilitate a combination of OH^−^ ions in the alkaline electrolyte for the OER. However, the high price and scarcity of resources restrict its wide application in energy conversion systems [13,14]. Therefore, cost-competitive catalysts with high catalytic activity urgently need to be explored.

Recently, earth-abundant and low-cost transition metal (TM)-based catalysts with improved catalytic OER stability have been an effective strategy for water splitting. Transition metal (i.e., Fe, Co, Ni, and Mo) oxides or hydroxides can form hydroxide intermediates during the OER process [15,16,17,18], but their high energy barrier and sluggish kinetics are still difficult to overcome due to fact of their poor conductivity [19,20,21,22]. Therefore, many efforts have been reported on the study of TM-based derivatives with modification of the surface electronic structure [23,24] including metal phosphides [25,26], sulfides [27,28], and fluorides [29,30]. Bimetal-based catalysts have the merits of enhanced reactivity and abundant active sites by the adjusted electronic structure at the metal/metal interfaces, which are important for improving the electrocatalytic performance [31]. Meanwhile, bimetal-based catalysts can form more oxygen vacancies and reduce the adsorption energy of anions in electrolytes [32]. As demonstrated by many reports, the incorporation of Fe^3+^ into transition metal-based catalysts can significantly increase the reactivity and catalytic activity during the OER process [33,34]. The iron–nickel bimetallic catalyst near the top of a volcano plot shows an excellent catalytic OER performance [35,36,37,38]. Importantly, the introduction of a conductive matrix in a bimetal system is favorable for increasing the electronic conductivity and catalytic stability, such as graphene [39], nickel foam [40], and amorphous carbon [41]. A couple of FeNi alloys with carbon supports can significantly increase the electrical conductivity, and the controllable construction of an FeNi/C hybrid can provide fast ion diffusion and enhance the electrocatalytic stability during the OER process.

Herein, we report iron–nickel/carbon (FeNi_3_/C) nanorods as an effective OER catalyst through hydrothermal and activation approaches. The nanorod morphology can increase the amount of exposed surface and the number of effective catalytic active sites, which are beneficial for increasing the electrochemical activity. The effect of the activation temperature on the crystallinity and catalytic OER behavior of the FeNi_3_/C nanorods were studied by physical characterization and electrochemical tests. As the optimal temperature was 400 °C, the FeNi_3_/C nanorods showed excellent OER performance. Only 250 mV of the overpotential was required at 10 mA cm^−2^ with a Tafel slope of 84.9 mV dec^−1^. The improved electrochemical stability was studied by chronoamperometry, which was indexed to the effect of rough morphology and optimal composition.

## 2. Experimental Section

### 2.1. Synthesis of the FeNi_3_/C Nanorods

A mixture of deionized (DI) water (12 mL) and ethylene glycol (36 mL), as the solvent to dissolve 200 mg of NiCl_2_⋅6H_2_O and 200 mg of FeCl_2_, and 200 mg of oxalic acid were subsequently slowly added under continuous ultrasonication. The solution was transferred to a stainless-steel autoclave (100 mL) and maintained at 150 °C for 12 h. As the temperature naturally cooled down, the precipitate was repeatedly washed with DI water/ethanol and dried at 60 °C under vacuum conditions overnight to obtain the FeNi nanorods. The FeNi nanorods were further thermally activated at 400 °C for 2 h with flowing N_2_ gas, and the target sample was named FeNi_3_/C. The FeNi_3_/C was thermally activated at 300 and 500 °C and labeled as FeNi_3_/C-300 and FeNi_3_/C-500. The related catalytic performances of FeNi_3_/C-300 and FeNi_3_/C-500 were compared. The synthetic process of the Fe nanorods and the Ni nanorods was the same as for the FeNi nanorods, and the precursor only included a single metal salt, either FeCl_2_ or NiCl_2_⋅6H_2_O. The samples were thermally activated at 400 °C, and the obtained powders were labeled as Fe/C and Ni/C nanorods for further use.

### 2.2. Characterization

The crystal structure was characterized by powder X-ray diffraction (XRD) (Bruker D8 Advance powder X-ray diffractometer, Cu Kα1, λ = 1.5405 Å, 40 KV, and 40 mA, Bruker, Saarbrucken, Germany). Scanning electron microscopy (SEM) images were obtained using an S-4800 II, Hitachi (Tokyo, Japan). The morphological structure was confirmed by transmission electron microscopy (TEM, Philips, TECNAI 12, Amsterdam, The Netherlands) and high-resolution transmission electron microscopy (HRTEM) (FEI Tecnai G2 F30 STWIN, 300 kV, FEI, Hillsboro, OR, USA). X-ray photoelectron spectroscopy was measured using a Thermo Science ESCALAB 250Xi (Thermofisher, Waltham, MA, USA).

### 2.3. Electrochemical Measurements

The electrochemical performance was performed using an electrochemical workstation (CHI 660E, Shanghai, China). The active material loaded on a glassy carbon electrode (GC, 3 mm diameter, 0.07 cm^−2^), graphite rod, and saturated calomel electrode (SCE) acted as the working, counter, and reference electrodes, respectively. The potentials were calculated to the reversible hydrogen electrode (RHE) by E(RHE) = E(SCE) + 0.0591 × pH + 0.24 V. The catalyst ink was prepared by mixing 5 mg of catalyst, 950 μL of ethanol, and 50 μL of Nafion solution (5 wt.% Nafion^TM^ in lower aliphatic alcohols and water, 15–20% water) under bath sonication. Then, the catalyst ink (10 μL) was dropped onto the GC and naturally dried. All data are presented with IR compensation unless otherwise noted.

The polarization curves were measured as the potential from 1.04 to 1.64 V vs. RHE at 5 mV s^−1^. Electrochemical impedance spectroscopy (EIS) was measured from 10^6^ Hz to 10^−2^ Hz. One thousand CV cycles were measured within the potential ranging from 1.04 to 1.64 V vs. RHE in 1 M KOH at a scan rate of 150 mV s^−1^, and a linear sweep was measured under a sweep rate of 5 mV s^−1^ after 1000 cycles. Chronoamperometry (CA) was tested at a voltage of 1.48 V for 15 h.

## 3. Results and Discussion

The iron–nickel alloy with conductive carbon (FeNi_3_/C) nanorods were synthesized via facile hydrothermal and thermal treatment methods. The high reducibility of ethylene glycol as the solvent could strongly couple the metal ions with oxalic acid, and the content of iron was accurately controlled to adjust the morphology of catalysts. After activation at a high temperature of 400 °C, the carbon ligands decomposed into carbon materials, which can improve the conductivity of the catalysts. Finally, the FeNi_3_/C nanorods were obtained.

**Figure 1:**
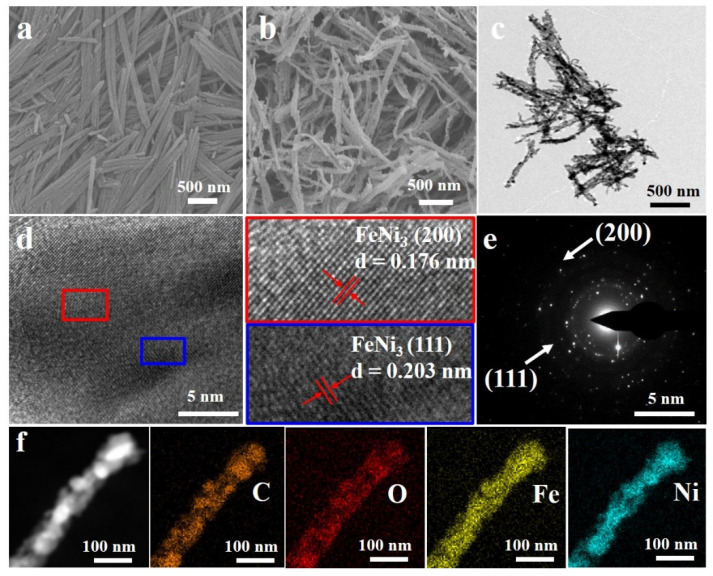
SEM images of (**a**) FeNi and (**b**) FeNi_3_/C nanorods; (**c**) TEM and (**d**) HR-TEM images of FeNi_3_/C nanorods; (**e**) SAED pattern; (**f**) STEM and elemental mappings of the FeNi_3_/C nanorods.

To probe the morphological structure of the FeNi_3_/C nanorods, scanning electron microscopy (SEM) was carried out. The FeNi nanorods were uniform and had an average length of 1.5 μm, as shown in Figure 1a, which was different from the Ni nanorods, which had irregular lengths, and the Fe nanorods, which had longer lengths of approximately 2 μm (Figure S1a,b). As a comparison to the precursors, the morphology of the FeNi nanorods was adjusted by the electrostatic interaction of metal ions, which is favorable for exposing abundant surface area. After thermal activation, the surfaces of the FeNi_3_/C nanorods became relatively rough due to the formation of the FeNi_3_ alloy and the decomposition of the carbon ligands (Figure 1b), which can provide abundant active sites. This morphology was further confirmed by transmission electron microscopy (TEM) images, as shown in Figure 1c. The average thickness of the nanorods was approximately determined to be 90 nm (Figure S2). Two d-spacings of 0.176 and 0.203 nm of the FeNi_3_/C nanorods corresponded to the (200) and (111) planes of the FeNi_3_ (Figure 1d). Figure 1e shows the corresponding selected area electron diffraction (SAED). The existence of concerned elements were found using energy-dispersive spectroscopy (EDS) (Figure S3a and Table S1), and the elemental mapping results confirmed that the distribution of the Fe, Ni, C, and O elements in FeNi_3_/C was uniform (Figure 1f).

**Figure 2:**
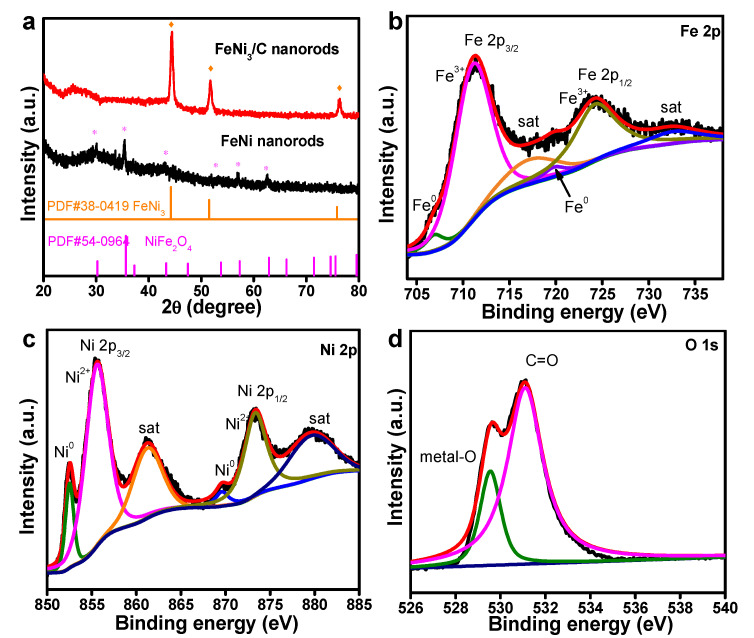
(**a**) XRD spectra of the FeNi and FeNi_3_/C nanorods (the asterisk and oranges squares represent the diffraction peaks of NiFe_2_O_2_ and FeNi_3_); high-resolution (**b**) Fe 2p, (**c**) Ni 2p, and (**d**) O1s XPS spectra of the FeNi_3_/C nanorods.

The change in the crystal structure of FeNi_3_/C was characterized by X-ray diffraction (XRD) (Figure 2a). The broadened peak at 25.8° corresponded to the (002) plane of graphitic carbon. The characteristic peaks for the FeNi nanorods were indexed to the existence of NiFe_2_O_4_ (JCPDS card No. 54-0964). In comparison, the FeNi_3_/C nanorods showed strong characteristic peaks at 44.2°, 52.0°, and 75.7^o^, owing to the (111), (200), and (220) planes of the FeNi_3_ alloy (JCPDS card No. 38-0419), which is agreement with the TEM results. The disappearance of the diffraction peaks of NiFe_2_O_4_ for FeNi_3_/C was attributed to the decomposition of metal oxides during thermal activation. Meanwhile, the effect of the annealing temperature on the crystallinity of the FeNi_3_/C nanorods was studied by XRD analysis (Figure S3b), and the average crystal size of the FeNi_3_/ nanorods was approximately 15.6 nm. As the activation temperature increased, the domain characteristic peaks of the FeNi_3_/C nanorods became stronger than for FeNi_3_/C-300, indicating the increased crystallinity due to the formation of FeNi_3_ alloys. There were no obvious changes in the characteristic peaks between FeNi_3_/C and FeNi_3_/C-500, demonstrating that the optimal temperature of 400 °C was high enough to form a stable catalyst.

The surface chemical circumstances of the FeNi_3_/C nanorods were probed by X-ray photoelectron spectroscopy (XPS), and C 1s at 284.8 eV was applied to standardize the binding energy. From the full scan of the XPS spectra, the FeNi_3_/C nanorods contained 11.6 atom% of O, 22.0 atom% of C, 19.5 atom% of Fe, and 46.9 atom% of Ni elements (Figure S4a). The C 1s spectra showed two dominant peaks at 284.8 eV for a C-C bond and at 288.6 eV for C-O bonds (Figure S4b). Figure 2b shows the deconvoluted Fe 2p spectra, and two distinct peaks were indexed to the spin-orbit coupling of Fe 2p_1/2_ and Fe 2p_3/2_ accompanying the satellite peaks. The peak was divided into Fe^0^ (706.9 and 719.8 eV) and Fe^3+^ peaks (711.3 and 724.8 eV), respectively [42]. The Ni 2p spectra were deconvoluted into Ni^0^ (852.5 and 869.7 eV) and Ni^2+^ (855.3 and 873.2 eV) with the related satellite peaks in Figure 2c [43], respectively. An energy difference of 17.8 eV was calculated between Ni 2p_3/2_ and Ni 2p_1/2_, implying that the Ni^2+^ state was dominant [44]. In addition, two peaks at 852.2 eV and 869.3 eV corresponded to Ni metal. The FeNi_3_/C nanorods with contents of Fe^3+^ and Ni^2+^ can act as active material, and the electrocatalytic behavior can be dramatically affected by the boosted active sites arising from the conversion of Ni^2+^ to Ni^3+^ during the OER process [45]. The deconvoluted O 1s spectra are shown in Figure 2d, and the two peaks at 529.5 and 532.3 eV corresponded to metal-O and C=O bonds [41]. The existence of metal-O bonds can probably be ascribed to the formation of oxidized states on FeNi_3_ alloy surfaces during thermal activation, and the internal high-oxygen coordination defects of the nanorods are generally considered as the dominant catalytic sites for increasing the oxidation kinetics and catalytic activity during the OER.

**Figure 3:**
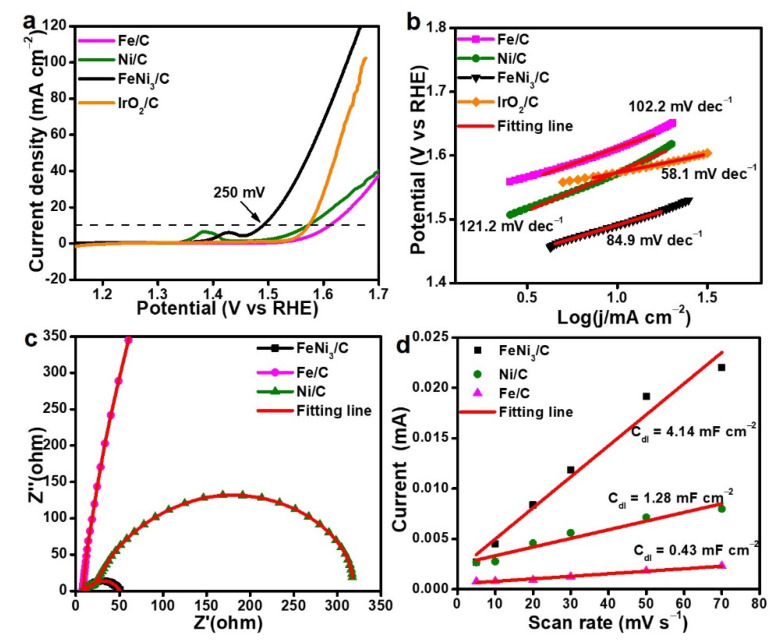
(**a**) Polarization curves of FeNi_3_/C, IrO_2_/C, Fe/C, and Ni/C nanorods at 5 mV s^−1^; (**b**) Tafel slopes of FeNi_3_/C, Fe/C, Ni/C, and IrO_2_/C; (**c**) Nyquist plots; (**d**) C_dl_ values of FeNi_3_/C, Fe/C, and Ni/C nanorods.

The electrocatalytic OER performance of the FeNi_3_/C nanorods was initially evaluated by cyclic voltammetry (CV) using a three-electrode configuration, and an aqueous 1 M KOH was the electrolyte with N_2_ purification. To reflect the effect of the activation temperature on the catalytic OER performance, the polarization curves of the FeNi_3_/C nanorods were compared (Figure S5a). To receive a current density of 10 mA cm^−2^, the overpotential of the FeNi_3_/C nanorods (250 mV) was smaller than 280 mV for FeNi_3_/C-300 and 290 mV for FeNi_3_/C-500, and the Tafel slope for the FeNi_3_/C nanorods (84.9 mV dec^−1^) was lower than 99.2 and 101.1 mV dec^−1^ for FeNi_3_/C-300 and FeNi_3_/C-500 (Figure S5b). As shown in Figure 3a, the FeNi_3_/C nanorods showed a lower overpotential at 10 mA cm^−2^ than that of Fe/C (370 mV), Ni/C (330 mV), commercial IrO_2_/C (327 mV), and other reported FeNi-based electrocatalysts for the OER (Table S2). The low overpotential of FeNi_3_/C implies a high OER activity due to the incorporation of Fe ions with Ni ions [46,47]. In addition, The Tafel slope can reflect the rate-determining step with the related reaction mechanism during the OER process, and Tafel slopes of 120, 60, and 40 mV represent the RSD of one-electron, chemical, and electron–proton reaction steps [48,49,50]. According to the Tafel slopes shown in Figure 3b, FeNi_3_/C had a smaller value of 84.9 mV dec^−1^ compared to 102.2 and 121.2 mV dec^−1^ for Fe/C and Ni/C, indicating faster catalytic kinetics for FeNi_3_/C. Chemical reactions with O_2_ formation as an intermediate on the catalytic sites was dominant for FeNi_3_/C, and the impact of the electron transfer process was no longer the primary step for the OER. The electrochemical dynamics and interfacial properties of the electrode were elucidated by electrochemical impedance spectroscopy (Figure 3c), and the calculated resistances are listed in Table S3 and were fit using Nyquist plots with an equivalent circuit in Figure S6. The charge transfer resistances (Rct) were 15.2, 110, and 26 Ω for the FeNi_3_/C, Fe/C, and Ni/C nanorods, respectively. The smaller Rct value indicates a faster charge transfer behavior as well as higher catalytic activity of FeNi_3_/C.

A catalyst exposing abundant active sites can show high electrochemical activity. The electrochemical surface area (ECSA) was estimated by CV measurement in a non-Faradic field (Table S4), and the double-layer capacitance (C_dl_) value was calculated by linearly fitting the current density versus scan rates (Figure S7). Specific activity was obtained by normalizing the origin current to the ECSA. The FeNi_3_/C nanorods with the optimal temperature of 400 °C had a specific activity of 0.24 mA cm^−2^ at the overpotential of 300 mV, which was higher than that of all control samples (Figure S8). The FeNi_3_/C nanorods had a C_dl_ value of 4.14 mF cm^−2^, as shown in Figure 3d, which was approximately 9.6 and 3.23 times higher than 0.43 and 1.28 mF cm^−2^ for Fe/C and Ni/C, respectively. This result confirms that FeNi_3_/C provided an enlarged catalytic active surface for facilitating ion diffusion and promoting the electrochemical reaction.

**Figure 4:**
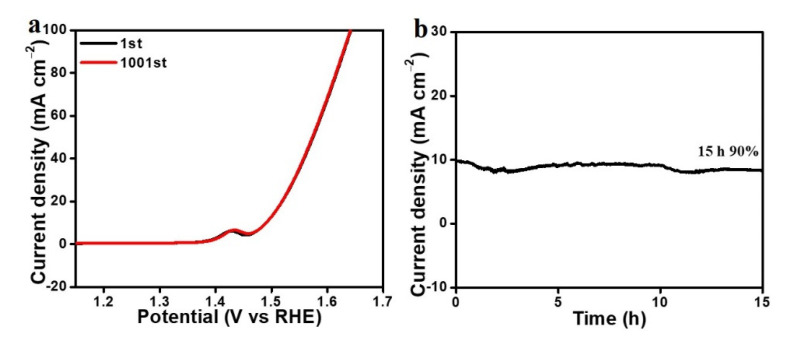
(**a**) The 1st and 1001st CV curves of FeNi_3_/C; (**b**) chronoamperometry test of FeNi_3_/C at the potential of 1.48 V.

The long-term stability of the FeNi_3_/C nanorods was initially evaluated by performing 1000 CV cycles, as shown in Figure 4a. The initial and 1001st CV curves almost overlap, and the overpotential at 10 mA cm^−2^ was a negligible change. Furthermore, chronoamperometry (CA) was measured at the potential of 1.48 V for 15 h (Figure 4b). The FeNi_3_/C nanorods exhibited no obvious change in current density at the initial 10 h, and the current density remained at 90% for the next 5 h. The Faraday efficiency of FeNi_3_/C was measured by comparing the experimental and theoretic amounts of oxygen gas produced during constant voltage electrolysis for 60 min (Figure S9), and the experimental volume was close to the theoretical oxygen volume, indicating that the oxygen evolution efficiency was close to 100%. These results demonstrate the outstanding electrocatalytic OER activity and stability of the FeNi_3_/C nanorods, attributed to the in situ formation of the FeNi_3_/C composites.

**Figure 5:**
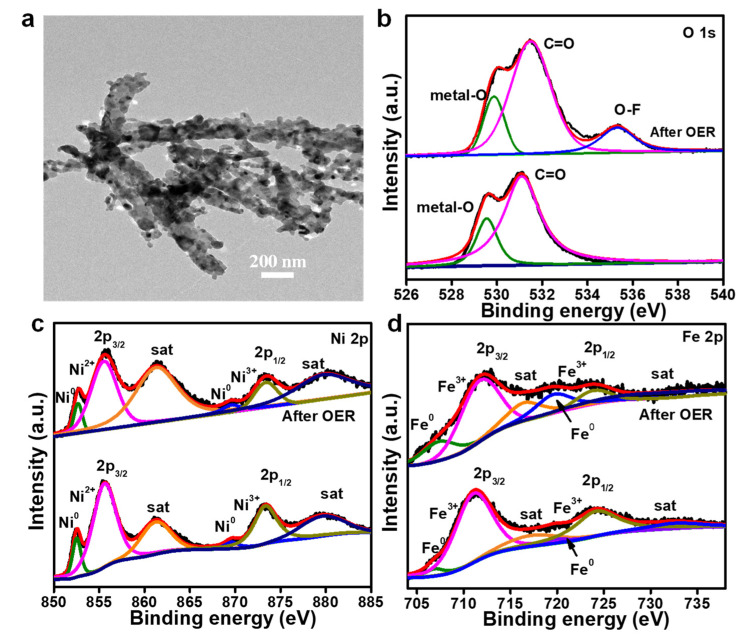
(**a**) TEM image of FeNi_3_/C after the stability test for the OER; (**b**–**d**) XPS spectrum of O 1s (**b**), Ni 2p (**c**), and Fe 2p (**d**) for the FeNi_3_/C alloy nanorod after the stability test for the OER.

The morphological change of the FeNi_3_/C surface after the stability test was characterized by TEM, as shown in Figure 5a. The morphology of FeNi_3_/C nanorods was maintained, and the slight collapse or fracture phenomena were caused by the partial oxidation of FeNi_3_ during the catalytic reaction in an alkaline solution. The change in the surface chemistry after the electrocatalytic test was confirmed by XPS. In comparison to the pristine state, the intensity of the metal-O bond after the CA test slightly increased with a shift of 0.4 eV because of the formation of intermediates. For the Ni element, the change in the Ni 2p spectra can be seen in Figure 5c, and the Ni 2p_3/2_ peak downshifted with by a value of 0.1 eV, attributed to the formation of nickel hydroxides or hydroxyl oxides after long-term CA testing. The Fe element showed a similar result, suggesting the formation of electroactive intermediates during the stability test, as shown in Figure 5d.

During the OER process, the high-valence nickel in the catalysts was more conducive to the rapid formation of intermediates (Ni-OH) in the electrolyte, which was further combined with the OH^−^ to form a nickel oxyhydroxide followed by the removal of oxygen. Therefore, the high content of Ni^2+^ in FeNi_3_/C was more conducive to the OER, and the adjusted surface electronic structure by incorporation of Fe^3+^ increased the absorbability of OH^−^, which resulted in the boosted catalytic activity of the catalysts during the OER process.

## 4. Conclusions

In summary, FeNi_3_/C nanorods as effective catalysts for the OER were constructed by combining the facile hydrothermal reaction and further thermal annealing treatment. The temperature and compositional effect on the catalyst were discussed. The FeNi_3_/C nanorods with an annealing temperature of 400 °C showed the best electrocatalytic performance such as a low overpotential of 250 mV at 10 mA cm^−2^, small Tafel slope of 84.9 mV dec^−1^, and high catalytic stability after CA testing for 15 h. The improved electrocatalytic behaviors were indexed to the controllable structure and optimal chemical composition by hybridizing bimetallic alloy with carbon. This work provides a strategy for preparing efficient catalysts for the OER by coupling bimetallic alloys with a carbon matrix.

## Data Availability

The data presented in this study are available on request from the corresponding author.

## Supplementary Materials

The following supporting information can be downloaded at: https://www.mdpi.com/article/10.3390/nano12152525/s1, Figure S1: SEM images of (a) Ni and (b) Fe nanorods; Figure S2: TEM image of FeNi_3_/C nanorods; Figure S3; (a) EDS data of FeNi_3_/C nanorods; (b) XRD patterns of FeNi_3_/C nanorods with different thermal annealing temperatures; Figure S4: XPS spectrum of FeNi_3_/C nanorods at (a) full scan and (b) C 1s; Figure S5: (a) Polarization curves and (b) Tafel plots of FeNi_3_/C nanorods with different thermal annealing temperatures; Figure S6: The equivalent circuit model of the EIS analysis of all samples; Figure S7: CV curves of (a) FeNi_3_/C nanorods; (b) Ni/C nanorods; (c) Fe/C nanorods at the potential of 1.04 V–1.14 V in 1 M KOH; Figure S8: (a) The specific activity of FeNi_3_/C at different activation temperatures; (b) the specific activity of FeNi_3_/C, Ni/C and Fe/C at the overpotential of 300 mV; Figure S9: Faraday efficiency of FeNi_3_/C for OER; Table S1: The atomic ratio of all elements from EDS; Table S2: The comparison of other FeNi-based OER catalysts in alkaline medium; Table S3: EIS fitting parameters from equivalent circuits for as-prepared catalysts; Table S4: The value of C_dl_ and ECSA for FeNi_3_/C with different annealing temperature. References [51,52,53,54,55,56,57,58,59,60] are cited in the Supplementary Materials.
