# Supplementary material for: Boosting the Oxygen Evolution Reaction by Controllably Constructing FeNi3/C Nanorods
Source: Nanomaterials (Basel). 2022 Jul 22;12(15):2525. doi: 10.3390/nano12152525 (PMC9332686; doi:10.3390/nano12152525)
Supplement: Supplementary file 1 [file nanomaterials-12-02525-s001.zip › nanomaterials-1791141-supplementary.pdf]

nanomaterials

MDPI

Supplementary Materials

Boosting the Oxygen Evolution Reaction by Controllably Constructing $FeNi_3$/C Nanorods

Xu Yu [1,*], Zhiqiang Pan [1], Zhixin Zhao [1], Yuke Zhou [1], Chengang Pei [1], Yifei Ma [2], Ho-Seok Park [3] and Mei Wang [2,*]

[1] School of Chemistry and Chemical Engineering, Yangzhou University, Yangzhou 225009, China; w461015600@163.com (Z.P.); zhao1zhi2xin3@163.com (Z.Z.); zykds66@163.com (Y.Z.); chengpyzu@163.com (C.P.)
[2] State Key Laboratory of Quantum Optics and Quantum Optics Devices, Institute of Laser Spectroscopy, Collaborative Innovation Center of Extreme Optics, Shanxi University, Taiyuan 030006, China; mayifei@sxu.edu.cn
[3] Department of Chemical Engineering, College of Engineering, Sungkyunkwan University, 2066 Seobu-ro, Jangan-gu, Suwon-si 440-746, Gyeonggi-do, Korea; phs0727@skku.edu
* Correspondence: yxypz15@yzu.edu.cn (X.Y.); wangmei@sxu.edu.cn (M.W.)

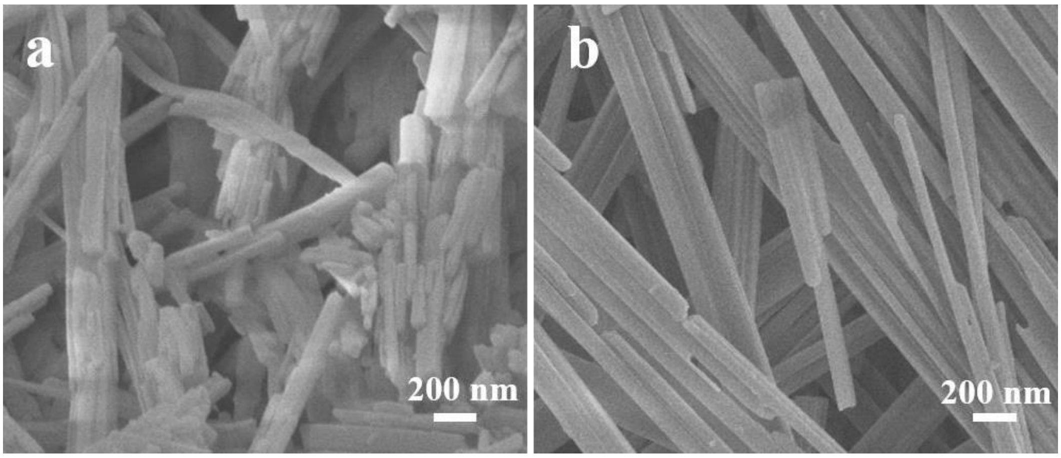

Figure S1. SEM images of (**a**) Ni and (**b**) Fe nanorods.

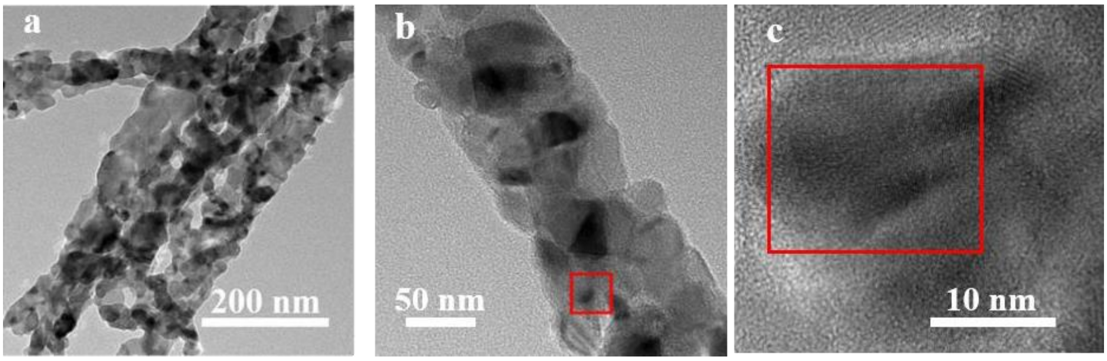

Figure S2. TEM images of $FeNi_3$/C nanorods at different magnifications.

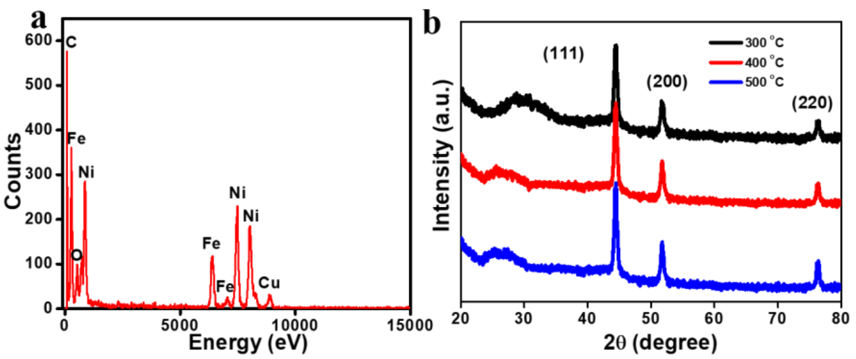

Figure S3. (**a**) EDS data of $FeNi_3$/C alloy nanorods; (**b**) XRD patterns of $FeNi_3$/C alloy nanorods with different thermal annealing temperatures.

The crystal size of the $FeNi_3$ alloys was calculated from XRD using the Scherrer equation, and the average size was approximately 15.64 nm.

$$D = \frac{K\lambda}{\beta\cos\theta}$$

where D is the crystallite size, K is the Scherrer constant, λ is the wavelength of the X-ray sources (0.15405 nm), β is the full width at half maximum, and θ is the peak position.

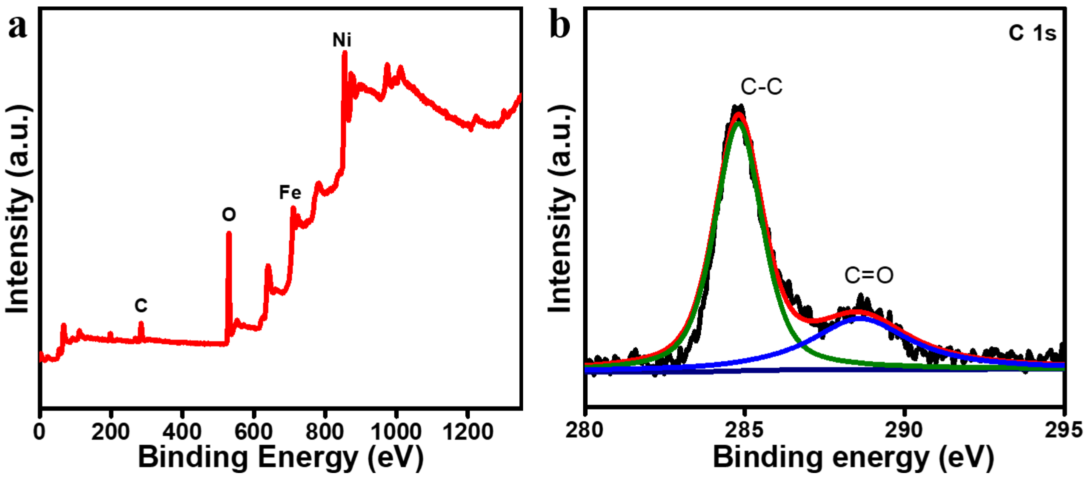

Figure S4. XPS spectrum of $FeNi_3$/C alloy nanorods: (**a**) full scan; (**b**) C 1s.

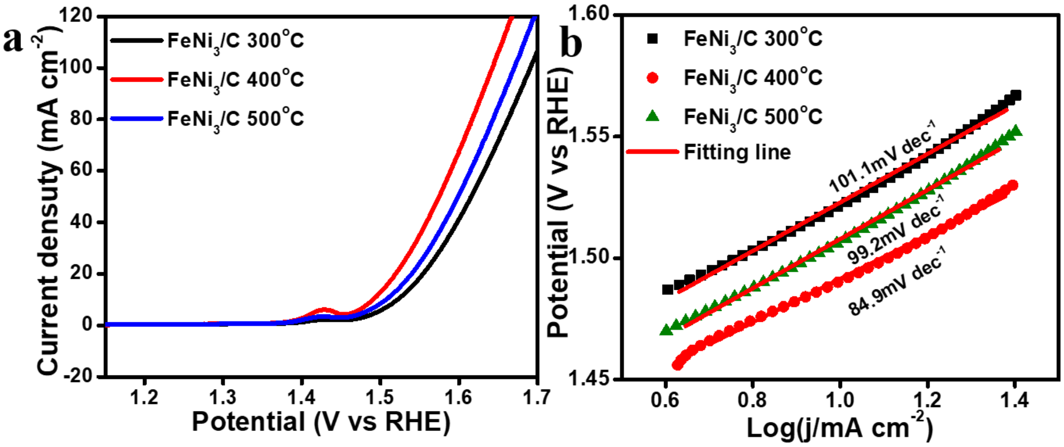

Figure S5. (**a**) Polarization curves and (**b**) Tafel plots of $FeNi_3/C$ alloy nanorods with different thermal annealing temperatures.

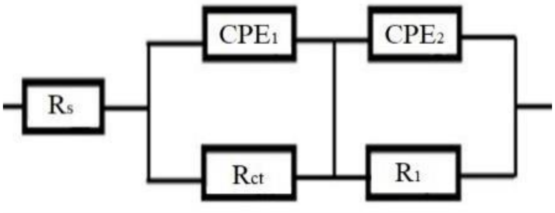

Figure S6. The equivalent circuit model of an EIS analysis of all the samples.

Figure S5. The fitting equivalent circuit model: R_s, is a sign of uncompensated solution resistance; R_{ct} isthe charge transfer resistance; R_1 is associated to the contact resistance between the catalysts and the othersresistance. The CPE was generally employed to well fit the impedance data by safely treating it as an empirical constant without considering its physical basis. Mostly, it was regarded as the double-layer capacitor from the catalyst/support and catalyst solution.

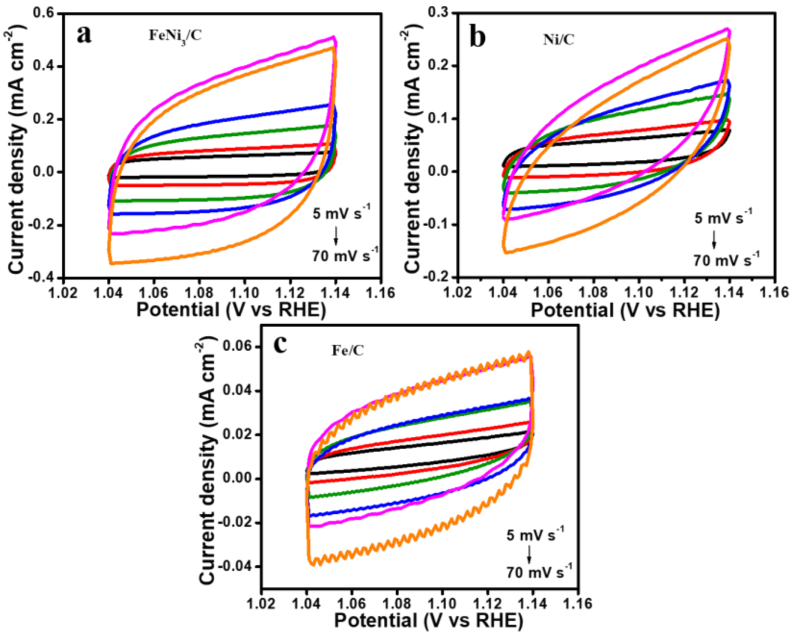

Figure S7. CV curves of (**a**) FeNi3/C alloy nanorods; (**b**) Ni/C nanorods; (**c**) Fe/C nanorodsat the potential of 1.04 V–1.14 V in 1 M KOH.

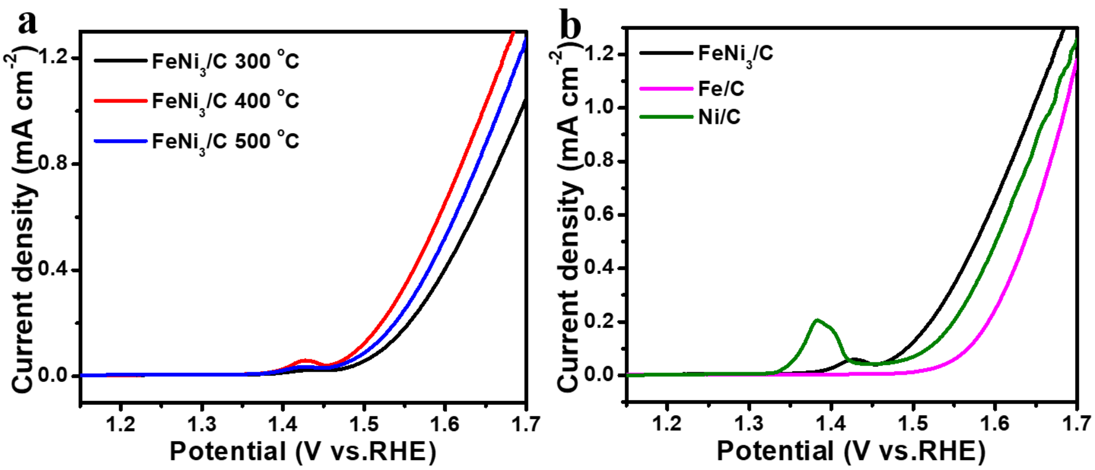

Figure S8. (**a**) The specific activity of FeNi3/C at different activation temperatures; (**b**)the specific activity of FeNi3/C, Ni/C, and Fe/C at the overpotential of 300 mV.

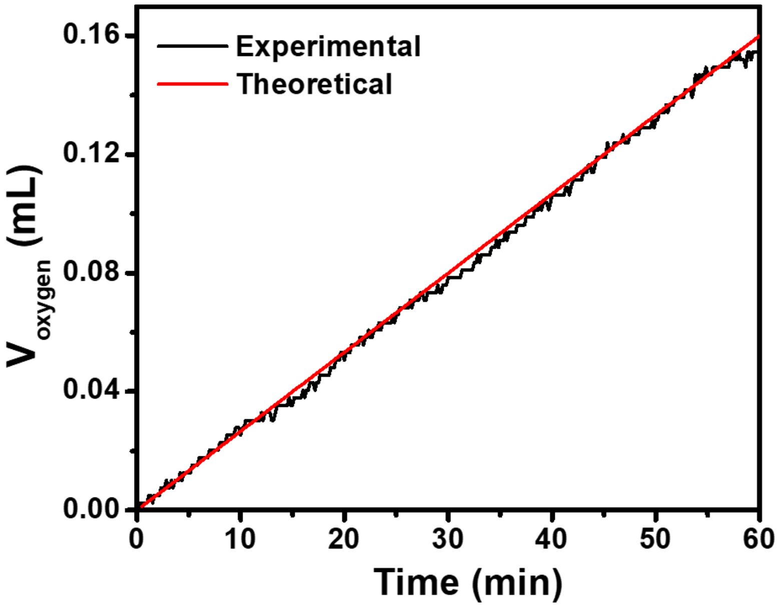

Figure S9. Faraday efficiency of $FeNi_3/C$ for the OER.

A gas-tight electrochemical cell with a gas burette was carried out to verify the faradaic yield of the sample using a typical three-electrode system. The working electrode was prepared by drop-casting catalyst suspension on a glassy carbon electrode with a surface area of 0.07 cm^{-2}. The graphite rod and saturated calomel electrode (SCE) acted as the counter and reference electrodes, respectively.To reach the benchmark current density of 10 mA cm^{-2} at approximately 1.48 V, the volume of the evolved gas was recorded synchronously. Thus, the faradaic yield was calculated from the ratio of the recorded gas volume to the theoretical gas volume during the charge passed through the electrode [1]:

$$\text{Faradic yeald} = \frac{V_{\text{experimental}}}{V_{\text{theoretical}}} = \frac{V_{\text{experimental}}}{\frac{1}{4} \times \frac{Q}{F} \times V_m}$$

where Q is the charge passed through the electrode; F is the Faraday constant (96485 C mol^{-1}); the number 4 represents 4 mole electrons per mole O_2; V_m is the molar volume of gas (24.5 L mol^{-1}, 298 K, and 101 KPa).

Table S1. The atomic ratio of all elements from the EDS.

C%	O%	e%	Ni%
24.3	10.2	17.8	47.7

Table S2. The comparison of other FeNi-based OER catalysts in alkaline medium.

Materials	Electrolyte	Overpotential (mV) at 10 mA cm^{-2}	Reference
$FeNi_3$/C alloy nanorods	1 M KOH	250	This work
FeNi-P/GA	1 M KOH	280	[2]
FeNi-LDH@3DG/CNTs	0.1 M NaOH	380	[3]
Ni-Co-F	1 M KOH	300	[4]
Graphene-Co_3O_4	1 M KOH	313	[5]
Fe_3O_4@Co_9S_8/rGO	1 M KOH	340	[6]
Ni-NiO-CNT	1 M KOH	320	[7]
Co_3O_4/mMWCNT	0.1 M KOH	390	[8]
NiOx/Ni	1 M KOH	390	[9]
Fe-Co-F-400	1 M KOH	250	[10]
NCG-CoO	1 M KOH	340	[11]

Table S3. EIS fitting parameters from equivalent circuits for as-prepared catalysts.

Sample	R_s/Ω	R_{ct}/Ω	R_1/Ω
$FeNi_3$/C	10.1	15.2	30.4
Ni/C	11.8	26.0	290.3
Fe/C	12.3	110.0	3000.0

Table S4. The value of C_{dl} and ECSA for $FeNi_3$/C with different annealing temperature.

Sample	C_{dl}/mF cm^{-2}	ECSA/cm^2
$FeNi_3$/C 300 ºC	1.86	3.25
$FeNi_3$/C 400 ºC	4.14	7.24
$FeNi_3$/C 500 ºC	2.47	4.32

Reference

1. Wang J.; Li K;, Zhong H.-X.; Xu D.; Wang Z.-L.; Jiang Z.; Wu Z.-J.; Zhang X.-B. Synergistic Effect between Met-al–Nitrogen–Carbon Sheets and NiO Nanoparticles for Enhanced Electrochemical Water- Oxidation Perfor-mance, Angew. *Chem. Int. Ed.* **2015**; *54*, 10530–10534.
2. Narendra Kumar A.V.; Li Y.; Yu H.; Yin S.; Xue H.; Xu Y;, Li X.; Wang H.; Wang L. 3D graphene aerogel sup-ported FeNi-P derived from electroactive nickel hexacyanoferrate as efficient oxygen evolution catalyst. *Electrochim. Acta* **2018**, *292*, 107–114.
3. Li Y.; Zhao M.; Zhao Y.; Song L.; Zhang Z. FeNi layered double-hydroxide nanosheets on a 3D carbon network as an efficient electrocatalyst for the oxygen evolution reaction. *Part. Part. Syst. Char.* **2016**, *33*, 158–166.
4. Xue, Y.; Wang, Y.; Liu, H.; Yu, X.; Xue, H.; Feng, L. Electrochemical oxygen evolution reaction catalyzed by a novel nickel–cobalt-fluoride catalyst. *Chem. Commun.* **2018**, *54*, 6204–6207.
5. Zhao Y.; Chen S.; Sun B.; Su D.; Huang X.; Liu H.; Yan Y.; Sun K. Wang G., Graphene-Co_3O_4 nanocomposite as electrocatalyst with high performance for oxygen evolution reaction. *Sci. Rep-UK* **2015**, *5*, 7629.
6. Yang J.; Zhu G.; Liu Y.; Xia J.; Ji Z.; Shen X.; Wu S. Fe_3O_4-decorated Co9S8 nanoparticles in situ grown on re-duced graphene oxide: A new and efficient electrocatalyst for oxygen evolution reaction. *Adv. Funct. Mater.* **2016**, *6*, 4712–4721.
7. Elizabeth I.; Nair A.K.; Singh B.P.; Gopukumar S. Multifunctional Ni-NiO-CNT composite as high performing free standing anode for Li ion batteries and advanced electro catalyst for oxygen evolution reaction. *Electrochim. Acta* **2017**, *230*, 98–105.
8. Lu X.; Zhao C. Highly efficient and robust oxygen evolution catalysts achieved by anchoring nanocrystalline cobalt oxides onto mildly oxidized multiwalled carbon nanotubes. *J. Mater. Chem. A* **2013,** *1*, 12053–12059.
9. Han G.-Q.; Liu Y.-R.; Hu W.-H.; Dong B.; Li X.; Shang X.; Chai Y.-M.; Liu Y.-Q.; Liu C.-G. Three dimensional nickel oxides/nickel structure by in situ electro-oxidation of nickel foam as robust electrocatalyst for oxygen evolution reaction. *Appl. Surf. Sci.* **2015**, *359*, 172–176.

10. Pei C.; Chen H.; Dong B.; Yu X.; Feng L. Electrochemical oxygen evolution reaction efficiently catalyzed by a novel porous iron-cobalt-fluoride nanocube easily derived from 3-dimensional Prussian blue analogue. *J. Power Sources* **2019**, *424*, 131–137.
11. Mao S.; Wen Z.; Huang T.; Hou Y.; Chen J. High-performance bi-functional electrocatalysts of 3D crumpled graphene–cobalt oxide nanohybrids for oxygen reduction and evolution reactions. *Energ. Environ. Sci.* **2014**, *7*, 609–616.
